# Local Government Stakeholder Perceptions of Legitimacy and Conflict of Interest: The Alcohol Industry and the "Drink Free Days" Campaign in England

**DOI:** 10.34172/ijhpm.2021.59

**Published:** 2021-06-19

**Authors:** Helen Walls, Benjamin Hawkins, Anna Durrance-Bagale

**Affiliations:** Department of Global Health and Development, Faculty of Public Health and Policy, London School of Hygiene and Tropical Medicine, London, UK.

**Keywords:** Alcohol, Alcohol Policy, Industry, Conflicts of Interest, England

## Abstract

**Background:** Industry involvement in alcohol policy is highly contentious. The Drink Free Days (DFD) campaign (2018- 2019) run by Public Health England (PHE), an executive agency of government, and Drinkaware, an industry-funded ‘alcohol education charity’ to encourage middle-aged drinkers to abstain from drinking on some days was criticised for perceived industry involvement. We examine the extent to which the DFD campaign was supported by local-authority Directors of Public Health (DPHs) in England – which have a statutory remit for promoting population health within their locality – and their reasons for this.

**Methods:** Our mixed-methods approach included a stakeholder mapping, online survey, and semi-structured interviews. The stakeholder mapping provided the basis for sampling survey and interview respondents. In total, 25 respondents completed the survey, and we conducted 21 interviews with DPHs and their local authority (LA) representatives. We examined survey responses, and coded free-text survey and interview responses to identify key themes.

**Results:** While some respondents supported the DFD campaign, others did not promote it, or actively opposed it, due mainly to concerns about conflicts of interest and the legitimacy of industry involvement in the campaign. These were considered to undermine PHE’s independence and deflect attention from more important, evidence-based policy interventions such as alcohol pricing while conferring vicarious credibility on Drinkaware. We also found low levels of knowledge about alcohol-related harm, the effectiveness of different policies to address these and the policy-influencing strategies used by the alcohol industry.

**Conclusion:** The findings highlight the dangers of industry partnership and potential conflicts of interest for government agencies and the ineffectiveness of the campaigns they run at local and national levels. They demonstrate the need for caution in engaging with industry-associated bodies at all levels of government and are thus of potential relevance to studies of other health-harming industries and policy contexts.

## Background

**Key Messages**

**Implications for policy makers**

- This study demonstrates the ways in which perceived conflicts of interest, arising from engagement with industry, may undermine the implementation of health policies and other initiatives at the local level.
- Policy-makers and implementers in national and local government should exercise caution when considering whether to partner, or otherwise engage, with health-harming industries in the development and delivery of policy measures.
- There is a need to address the often low levels of knowledge among policy-makers working in the area of alcohol and health, with regard to the nature of alcohol-related harms, the effectiveness of different types of policy to address these, as well as the strategies used by the alcohol industry to influence such policy-making.
- Policy-makers at all levels of government need to develop a more nuanced understanding of industry strategy and the ways in which influence may be exerted even when such actors present themselves as offering solutions to policy problems.

**Implications for the public**

The involvement of industry actors in the development and implementation of alcohol and other health policies is highly contentious. Public Health England’s (PHE’s) partnership with Drinkaware in the Drink Free Days (DFD) campaign provides a cautionary tale for policy-makers and implementers in national and local government faced with dilemmas about whether to engage with industry-funded bodies in the development or delivery of such measures. Industry participation in public health campaigns can often appear benign, but influence over policy often occurs in indirect ways. This may detract attention from other, more effective measures (eg, pricing increases or advertising restrictions), or shape the content of public messaging campaigns such as DFD. It is important, therefore, to be aware that Drinkaware is principally funded by the alcohol industry and how this may affect the issues they prioritise and the solutions they propose, as well as the effectiveness of government activities. Additionally, government agencies should be cautious in engaging such industry partners.

From 1980 to 2014 there was a 60% increase in the affordability of alcohol in the United Kingdom, leading to increased population-level alcohol consumption and an increase in alcohol-related harm.^1,2^ This has been particualrly pronouced in the off-trade sectors and coincided with a shift in consumption patterns away from pubs and on-trade premises to home drinking. The affordability of beer in supermarket and off-licences, for example, rose by 188% and wine and spirits went up by 131% between 1987 and 2018.^1^ At the same time, there has been a significant increase in alcohol-related harm including a 400% rise in liver disease since the 1970s.^2^ This has given rise to highly contested policy debates regarding how best to address the health and social impacts of alcohol and the legitimate role of the alcohol industry in policy development and implementation of alcohol policy.^3-6^ This includes debates about the role of industry social aspects organisations such as the UK’s Portman Group. These are arms-length bodies established, funded and/or controlled by the alcohol industry, which engage with issues surrounding alcohol’s societal effects.^7,8^

 The ‘alcohol education charity’ Drinkaware was established by the Portman Group in 2004, before being spun off as a separate entity in 2006 via a memorandum of understanding between the Departments of Health in Westminster and the devolved administrations and the Portman Group. The new Drinkaware organisation was based in the same building as the Portman Group.^9,10^ Reflecting its origins, its board initially included equal representation from the public health community and the alcohol industry.^9^ Following a 2013 review,^11^ Drinkaware’s management structure was reformed, removing industry representation from the governing board, and Drinkaware now occupies independent premises. Nonetheless, Drinkaware remains controversial for many public health actors due to its ongoing dependence on the alcohol industry for over 90% of its funding, which is seen to create at least a potential conflict of interest (COI). The latter refers to a situation whereby the commercial or other interests of actors are affected by policy regimes in ways that make it inappropriate for them to be involved in the development and/or delivery of those policies.^9,12,13^

 Controversy surrounding Drinkaware’s role in policy delivery came to the fore in 2018 in relation to the Drink Free Days (DFD) campaign run in association with Public Health England (PHE); an executive agency of the Department of Health and Social Care, tasked with the promotion of population health in England. The DFD campaign sought to encourage drinkers aged 40-64 to abstain from drinking on certain days in line with the Chief Medical Officer’s revised 2016 drinking guidelines.^14^ These guidelines state that men and women should not drink more than 14 units per week, and these should ideally be spread over the week, with individuals taking several drink-free days each week. Regular drinking can increase the risk of disease, including certain cancers, even when drinking fewer than 14 units weekly. Risks of acute injury increase 2- to 5-fold after drinking 5-7 units over a period as extended as 6 hours.^14^ In keeping with similar health messaging campaigns run by PHE, it was expected that local authority (LA) public health teams and Directors of Public Health (DPHs) – which have a statutory remit for promoting population health within their locality in England – would promote the campaign and use the associated materials in the service provision.

 The PHE-Drinkaware co-branded campaign consisted of a website, mobile phone applications, radio and digital media campaign as well as supporting materials for LA public health actors to download and use. It ran in two waves from September to November 2018 and February to March 2019, and received extensive criticism from public health actors.^15^ The partnership between PHE and Drinkaware led to concerns among some public health actors that the campaign may have proven ineffective or may even have undermined public health objectives. PHE thus undertook an independent review of the campaign to examine support among key stakeholders (ie, LA DPH) from which this study is drawn.

 This article examines the extent to which, why and how, the DFD campaign was supported by local government public health actors and their perceptions of the campaign. In particular, it investigates how the controversial partnership between a government agency and an industry body affected the implementation of the campaign. While the scope of the article is limited to alcohol policy in England, the issues raised here about the effects of industry engagement, the perceptions of LA policy actors and the connections between central and local government on the effectiveness of health policies and programmes are applicable to other issues and policy contexts in which similar partnerships with controversial industries are considered.

## Methods

 We undertook a mixed-methods research approach involving the following steps: (1) stakeholder mapping; (2) online questionnaire-based survey; (3) semi-structured in-depth interviews. The stakeholder mapping^16^ proceeded through analysis of PHE’s website and related materials, LA websites of English DPHs, professional associations (eg, the British Medical Association) and health- and alcohol-focused non-governmental and civil society organisations (eg, Royal Society of Public Health) to identify relevant policy actors including LA DPHs in England. This provided the basis for the purposive sampling of respondents for the survey and semi-structured interviews. We identified 134 DPH posts across the 152 LAs in England, with 118 individual DPHs currently in post at the time of the study.

 The online questionnaire survey was designed using a survey tool provided by the host university [redacted to facilitate blind peer review]. Requests to complete this were sent via email to the 118 DPHs identified in the stakeholder mapping, on November 12, 2019. Follow-up emails were sent periodically and finally telephone calls were made to potential respondents in the period before the survey closed on December 15, 2019. In total, 25 respondents completed the survey, giving a response rate of 21%.

 The questionnaire consisted of 29 questions organised around 4 key themes: (1) the respondents’ role in the DFD campaign; (2) the development of the DFD campaign; (3) the respondents’ views on the DFD campaign; (4) the respondents’ views on Drinkaware. Some questions were designed using a 5-point Likert scale, while others requested free-text responses. All respondents were anonymous unless they indicated their willingness to participate in a follow-up interview. Consequently, we do not differentiate between DPHs and other LA public health actors in reporting the survey results. Questionnaire responses were collated and analysed by calculating the frequency of particular responses for closed questions and through manual qualitative coding of the free-text responses. Given the rudimentary quantitative analysis undertaken, and the impossibility of ensuring a fully representative sample in a small ‘n’ study such as this, no specific statistical software was employed. The quantitative data are designed to give a descriptive flavour of the responses rather than forming the basis for more robust statistical inferences. The key themes identified were used to inform the development of the interview protocol.

 A total of 21 interviews were conducted with DPHs and other LA public health representatives (ie, members of the LA public health team working under the DPH) in person or via telephone. Respondents were identified from our stakeholder mapping and from snowball sampling (ie, suggestions by interviewees).^17-19^ We interviewed at least two DPHs or other LA public health actors from each of the eight participating regions of England (the additional region, the South-West, did not participate in the DFD campaign).The interviews lasted between 15 and 60 minutes, with most around 30 minutes. Of the 21 interviews conducted, 18 were voice recorded as some respondents declined recording. Where recording was not permitted, detailed notes were taken by the interviewer.Recorded interviews were reviewed for relevance on the basis of the degree of engagement of the respondent with the DFD campaign and their ability to provide information about or opinions on this. Sixteen interviews were transcribed and thematically coded using NVivo 10 data analysis software.^20^ The codebook was developed inductively and agreed after independent review of transcripts by two analysts before formal coding in NVivo 10. Interview notes from non-recorded interviews were then reviewed for any differences in terms of key themes and findings and no significant variations were identified. In order to ensure accuracy, all quotations are taken from the transcribed interviews and we have not reproduced the responses of non-recorded interviews on the basis of interviewer notes. The quotations included are selected as examples of the points raised in the analysis below as not all relevant quotes can be included. However, they are selected on the basis that they are representative of wider views or trends within the overall data set analysed.

 Interview respondents were provided with information sheets and consent forms via email prior to the interview and asked to give informed consent by competing the form before being interviewed. Usually this was done on the day immediately before the interview. On the form they were asked to indicate how their responses could be used by researchers and attributed in publications and other study outputs. The study was approved by the [redacted university] ethics committee. In reporting the interview responses, we differentiate between DPH and LA public health actors since transcripts were anonymised and coded according to these categories. We do not however draw any analytical distinctions between these categories.

## Results

###  Respondent Engagement With the Campaign

 The degree to which LA representatives (including DPHs) supported the DFD campaign varied significantly. More than half (52%) of respondents to the survey questionnaire reported that they did not promote the campaign. Of those respondents (44%) who promoted the campaign, the most common promotional methods involved liaising with local healthcare service providers, posting on social media, carrying details of the campaign on the LA website, and having the campaign material on LA premises (eg, local council buildings) (Table 1).

**Table 1:** Promotion of the Campaign by Survey Questionnaire Respondents

**Question Asked**	**Response Types**	**No. (%)**
Did you promote the DFD campaign?	Yes	11 (44)
	No	13 (52)
	Don’t know	1 (4)
How did you promote the DFD campaign?	Liaised with local providers	7 (35)
	Social media	5 (26)
	Promoted it on the LA website	4 (21)
	Campaign material on LA premises	2 (11)

Abbreviations: LA, local authority; DFD, Drink Free Days.

 The in-depth interviews helped to further clarify respondents’ roles and engagement with the campaign. From these interviews, we identified three types of respondents (Table 2). Approximately a third of the in-depth interview respondents, whom we term ‘proponents,’ articulated strong support for the campaign. These actors undertook promotional activity for the campaign within their usual communication channels, such as LA public health websites, social media accounts and local area healthcare service providers:

*“[Drink free days] is something that we do promote throughout our work, so we very much support it [the campaign]” *(LA respondent, 11).

**Table 2:** Type of Response to the Campaign by In-depth Interview Respondents

**Respondent Groupings**	**No. (%)**
Proponents	6 (38)
Tacit supporters	4 (25)
Principled opponents	3 (19)
Other	3 (19)

 At the other end of the spectrum was strong opposition to the campaign, which led LAs to take a clear decision not to support the campaign and, in many cases, to articulate clearly that they would not be doing so. We label this group, who represent about a fifth of respondents, ‘principled opponents:’

*“I absolutely wouldn’t work with [Drinkaware], and I certainly wouldn’t have promoted the PHE Drinkaware campaign because it looks like they’re endorsing Drinkaware as an organisation. […] we were just horrified at the fact that PHE would be prepared to collaborate with such an organisation […] So we didn’t do anything to promote it at all. And actually we wrote to all of our colleagues in the system and asked them not to promote it too” *(DPH respondent, 4).

 Other actors expressed some reservations about the campaign but did not explicitly oppose it. However, while they formally supported the campaign, their support can be described as lukewarm. To the extent that they promoted the campaign this was largely passive (ie, linking to the campaign on LA website), not involving any active promotional activities or campaigning. We term this group, who made up about a quarter of respondents, ‘tacit supporters:’

*“We didn’t actively come out in opposition to the campaign, we just didn’t actively support it… So we didn’t actively promote it, but we didn’t make a huge thing of it. Obviously some of our colleagues did. We just kind of kept it fairly low key, we did not actively promote the campaign”* (DPH respondent, 3).

 A final group, who were about a fifth of respondents, did not fall clearly within any of the groups. These were respondents demonstrating low levels of engagement with the campaign.

###  Views of Drinkaware

 In the responses to the survey questionnaire, almost half of respondents (48%) considered Drinkaware to be an ‘alcohol industry body,’ with 24% considering it a ‘government-industry partnership,’ 12% describing it as an ‘independent health charity’ and 4% as a ‘government agency.’

 Opinions were fairly evenly split with regard to whether Drinkaware has a legitimate role to play in providing public health information such as the DFD campaign. When asked about Drinkware’s role in general terms, 40% of respondents reported that Drinkaware has a legitimate role to play in public health information, while 36% disagreed with this (with 24% of respondents selecting ‘strongly disagree’). However, respondents were more clearly opposed to the involvement of Drinkaware in the DFD campaign specifically. Forty percent (40%) of respondents said that it was not legitimate for PHE to partner with Drinkware on the campaign, while 24% said that it was legitimate. Similarly, more respondents (44%) thought that PHE should not undertake future campaigns in partnership with Drinkaware than thought PHE should do so (28%; Table 3).

**Table 3:** Understandings of Drinkaware by Survey Questionnaire Respondents

**Question Asked/Statement Proposed**	**Response Types**	**No. (%)**
Which of the following best describes Drinkaware as an organisation?	Alcohol industry body	12 (48)
	Government-industry partnership	6 (24)
	Independent health charity	3 (12)
	Other	2 (8)
	Government agency	1 (4)
	Don’t know	1 (4)
Drinkaware has a legitimate role to play in public health information such as the DFD campaign	Agree	10 (40)
	Neither agree nor disagree	6 (24)
	Disagree	9 (36)
	Don’t know	0 (0)
It is legitimate for PHE to partner with Drinkaware on the DFD campaign	Agree	6 (24)
	Neither agree nor disagree	9 (36)
	Disagree	10 (30)
	Don’t know	0 (0)

Abbreviations: PHE, Public Health England; DFD, Drink Free Days.

 Respondents who considered Drinkaware to be an ‘independent health charity’ were more likely to have promoted the campaign (two thirds promoting it, and one third not promoting it), compared with people who considered it a ‘government-industry partnership’ (only half of whom promoted it). Those who considered it an ‘alcohol industry body’ were the least likely to promote it (one third promoting it, and two thirds not promoting it).

 The ‘principled opponents’ group identified Drinkaware as an industry body and as such saw a partnership with PHE – a public entity, tasked with the promotion and maintenance of population health – to be inappropriate and representing a COI. Within the overall concerns about the association between Drinkaware and PHE – undermining the perceived independence of PHE while conveying vicarious credibility on Drinkaware – some actors were particularly concerned about the impact of the campaign on PHE’s *One You* brand (under which they run wider public health and lifestyle messaging campaigns), which featured on the campaign materials alongside Drinkaware’s logo. There were concerns also that the Drinkaware campaign would be ineffective and deflect attention from more important, evidence-based interventions such as those around pricing. In addition to the general reservations about the effectiveness of relying on public information campaigns and individual behaviour change in isolation from whole population measures, some respondents believed the level of resources available was inadequate, particularly in light of the reputational damage to PHE and the divisions created between public health actors:

*“In the past, there have been some examples of where the messages that they [Drinkaware] were giving, some of the campaigns that they’ve done have not been effective and, in fact, have worked the opposite way. So, I’m sceptical about their materials now and I don’t really trust them. Happy for them to give money to the government to fund campaigns, but not for them to be involved in the board or directing how those campaigns work, or the focus of them, or the messages. And I’m not satisfied that that happens now, so, hence I would avoid using them”* (LA respondent, 10).

 In the context of post-austerity budget cuts, and the increasing demands placed on LAs and associated public services, some respondents felt they simply could not ignore any additional resources or campaigns regardless of their governance or funding. They feared that the alternative to promoting the DFD campaign was that there would be no campaign at all and that this would have a net negative effect on public health.

 Interestingly, the alcohol industry was frequently differentiated from the tobacco industry, with whom respondents felt it would be inappropriate to engage, while comparisons were also drawn frequently with the gambling industry as a problematic sector with which to engage. Alongside arguments that industry engagement is necessary, some respondents in this group stated that they did not see Drinkaware as part of the alcohol industry, and thus the calculation about whether to engage with them would be different to engaging with an alcohol producer or industry body directly. In some cases, respondents articulated these views concurrently.

*“I personally find some of the stuff they [Drinkaware] produce quite useful, particularly some of the stats and the research and stuff” *(LA respondent, 1).

*“I think it’s a really good organisation. I think that the tools and the resources are really good and […] that everyone is widely aware of being able to access those resources for campaigns and things like that” *(LA respondent, 6).

*“I think we’ve still got a little bit of convincing that Drinkaware is not, is disassociated from the alcohol *industry *[…]. But having said that, I don’t think it’s impossible to work as long as you have to be fully confident of the situation, of your partner and just be clear that […] there isn’t any influence to try and undermine the message […] what our agenda is, what our programme is”* (LA respondent, 4).

###  Reasons for Supporting or Opposing the Campaign

 Overall, the campaign was considered by survey respondents to be appropriate in terms of its content and messaging on alcohol-related harms, which were considered to reflect the Chief Medical Officer’s 2016 drinking guidelines.^14^ A greater proportion of respondents considered that the campaign was likely to help people modify their drinking (29%) than believed it would not (21%), while 33% were unsure if the campaign would have an impact.

 Free text responses to the survey questionnaire and in-depth interviews identified a number of reasons for supporting or opposing the campaign, including: Drinkaware’s involvement in the campaign; their capacity to undertake health promotion work; the local relevance of the campaign; and the poor communication of the campaign to stakeholders and thus lack of lead time to prepare. Of these the most frequently cited reason was the involvement of Drinkaware.

 When asked, in the survey questionnaire, to indicate the extent to which they agreed with several statements, 20% of respondents said that involvement of Drinkaware was a factor in their decision about whether to promote the DFD campaign or not. Four out of five respondents in this category did not support the DFD campaign. Against this, 36% of respondents said that this was not a factor, with 44% in either the ‘neither agree nor disagree’ or ‘don’t know’ categories (see Table 4). The opposition of public health actors to Drinkaware’s involvement was also cited as a factor in their decision by 20% of respondents (none of these supported the campaign), while 28% said that this was not a factor in their decision (with a combined 52% falling in the neutral and don’t know categories). Four of the respondents agreed or strongly agreed with both statements.

**Table 4:** Effects of Drinkaware’s Involvement in the Campaign on Survey Questionnaire Respondents

**Statement Proposed**	**Response Type**	**No. (%)**
The involvement of Drinkaware was a factor in our decision of whether to promote the DFD campaign	Agree	5 (20)
	Neither agree nor disagree	8 (32)
	Disagree	9 (36)
	Don’t know	3 (12)
The opposition of public health actors to the involvement of Drinkaware was a factor in our decision of whether to promote the DFD campaign	Agree	5 (20)
	Neither agree nor disagree	10 (40)
	Disagree	7 (28)

Abbreviation: DFD, Drink Free Days.

 Among interviewees, support for the DFD campaign was also limited because they did not want to replicate or detract from local campaigns. Related to this, many LAs have limited budgets, multiple competing priorities and have a predetermined calendar of activities throughout the year. There appeared to be little willingness or capacity to undertake additional alcohol-related activities beyond these. As exemplified in the quote below, several respondents felt the message of taking drink-free days was too weak and potentially problematic as it undermined the need for individuals to monitor and limit aggregate weekly consumption:

*“I think the other thing as well, which is an unfortunate side effect of the message […], is the fact that it might inadvertently legitimise drink days. Someone could say well you’ve done two drink free days does that mean I can have five drink days? You can turn it on its head and that’s a worrying side *effect” (LA respondent, 1).

 Similarly, local support was affected by a perception that PHE did not promote the campaign adequately and thus it was not sufficiently visible. The lack of forewarning about the campaign and the lack of opportunity to feed into, or express reservations about, its design and execution was also a factor in the controversy which emerged around it identified by interviewees. However, while earlier engagement may have mitigated some of the criticisms which were articulated about the partnership, respondents did not feel that this would have fully resolved the issues. Those in the ‘principled opponents’ category, at least, saw engagement with what they perceived to be an industry body as a clear red line.

 Many of those in the ‘tacit supporters’ camp shared some of the reservations articulated by the ‘principled opponents’ about the involvement of Drinkaware in the DFD campaign (and in public health messaging more generally), but either did not feel strongly enough about this to oppose the campaign or took what they saw as a more pragmatic approach toward their situation and the resources available:

*“I’m probably more towards the pragmatists. I’m consistently frustrated by our lack of resource and ability to do anything and, I think, sometimes, you know, these alcohol companies have a social responsibility so let’s just utilise that”* (LA respondent, 2).

 Some respondents in the ‘tacit supporter’ category held what could be considered contradictory ‘principled’ and ‘pragmatic’ positions on Drinkaware. While some respondents expressed reservations about Drinkaware’s legitimacy as a policy actor, in some instances referring to potential COI and commercial imperatives, these were overridden by practical considerations and the potential to offer relevant campaigns in the context of limited resources. As one DPH stated, they would be open to potential future collaborations with Drinkaware:

*“Having [said] twenty minutes ago I wouldn’t touch Drinkaware with a bargepole, yeah, I probably would. […] Whatever my reservations about Drinkaware and my perception of the public health relationship being tainted, if they were to come out locally and it would have enabled me to get some resource into broader media comms [communications] messaging, linked to then putting forward a message in a way that I was more comfortable with” *(DPH respondent, 2).

 While ‘proponents’ of the campaign recognised some of the pragmatic and resource-related limitations on promoting it, they did not share the reservations articulated by ‘tacit supporters’ about the involvement of industry actors in the delivery of public health messaging like the DFD campaign. The rationales offered for their positions were largely couched in pragmatic terms: that the industry is such a significant and sizeable actor in the field that it simply cannot be ignored or excluded from the policy sphere, regardless of the reservations some may have about the COI. Some respondents argued that it would be undesirable to exclude industry given their resources and expertise, and would result in sub-optimal policy and health outcomes. Some respondents referred to successful partnerships between the government and PHE with industry actors in other sectors, such as the food and even the gambling industry, which they felt has had a positive impact on public health. As a result of this, they thought the same approach could be extended to alcohol.

 Two other themes identified in the interviews also relate to respondent support for or opposition to the campaign. Firstly, levels of knowledge about the nature of alcohol-related harms and alcohol policies were low among many respondents and their knowledge, or at least recall, of the DFD campaign was also limited. This emerged in interviews as a lack of familiarity with the most effective, evidence-based policy measures in the international research consensus and the need for ‘upstream’ protective measures as well as the wider debate on industry involvement in these processes.^3^ Secondly, and in contrast to the previous point, levels of awareness of, and engagement with, alcohol policy topics was in the North East region very high. This is reflected in the response rate to interview requests and the insights into issues associated with alcohol industry engagement in policy-making which interviewees from this region demonstrated. Moreover, there appeared to be a degree of co-ordination and agreement about the response to the DFD campaign, and the partnership, among public health actors in this region. Respondents explained that this reflected both the need to tackle high levels of alcohol-related harms in this region as well as the existence of strong regional public health institutions and structures. These included the regional PHE office and regional forums for DPHs which facilitate an ongoing exchange of ideas between different LA actors. Similarly, the existence of an alcohol-focused organisation such as Balance North East, commissioned and funded by the different LAs across the region, means they are able to maintain a high level of engagement on alcohol policy issues more specifically. This was in contrast to other LA respondents, who discussed limited resources for alcohol issues and competing demands from multiple public health priorities.

## Discussion

 This study aimed to examine the degree of support for the DFD campaign and the reasons for this among LA public health actors. While some LA public health actors supported the DFD campaign, strong opposition to the campaign expressed by others was due, principally, to concerns about the perceived COI arising from PHE’s partnership with what these respondents saw as an alcohol industry body. This was felt to undermine PHE’s independence and deflect attention from more important, evidence-based policies while conferring vicarious credibility on Drinkaware, as evident in previous studies.^21,22^ A third group of respondents took a more equivocal position. While they expressed reservations about Drinkaware’s legitimacy as a policy actor and the potential for COIs, they saw the campaign as worthwhile in the context of limited public health resources. This suggests a reductionist conception of COI and corporate political strategy, which fails to recognise the indirect and long-term nature of much policy-influencing activity. These strategies include activities designed to shape the policy environment and build relationships with policy actors through the delivery in the context of constrained government resources.^23^

 Proponents of the campaign offered largely pragmatic reasons for supporting and promoting it: that the industry is able to supply otherwise scarce resources to undertake public engagement work which otherwise would not occur. This suggests that austerity policies offer industry actors an opportunity to engage policy actors keen for resources. It reflects also the contestable assumption by LA actors that industry-funded campaigns are effective rather than counterproductive.^24,25^ In addition, it was felt by some respondents that such a significant actor simply cannot be ignored or excluded from the policy sphere, regardless of reservations about potential COI. This argument had both normative as well as practical components with some respondents arguing that it would be undesirable to exclude industry given their resources and expertise, and would result in sub-optimal policy (and health) outcomes. Analyses of corporate strategy have identified attempts by industry to frame the terms in which policy debates are conducted, and the views of these respondents reflect the type of issue framing which the alcohol industry seeks to perpetuate.^21,26,27^ This reflects the extent to which public discourses on alcohol-related harms and policy approaches are often structured in terms amenable to industry-favoured approaches.^26^

 We also found generally low levels of knowledge about the nature of alcohol-related harms, the effectiveness of different types of policies to address these and the strategies used by the alcohol industry to influence these among LA respondents working in the area of alcohol and health. This may have led some LA actors to underestimate the potentially negative effects of industry-associated campaigns which seemed to provide additional resources. Industry-funded bodies may engage with programmes such as the DFD campaign which directly coincide with the work of these LA actors as part of their wider corporate social responsibility strategies, including funding bodies such as Drinkaware.^13,21,28^ These are rarely effective but may have a policy displacement effect, detracting attention from other, more impactful measures.^24,25,29^ A higher level of awareness about the issues raised by industry engagement and the often indirect, and apparently benign, ways in which industry actors may seek to engage and thereby compromise LA actors is needed. Given the relatively high awareness of the issues raised here and the identification with alcohol-related issues more generally in the North East there may be the potential for policy learning and shared practice with other regions.

 Drinkaware’s involvement in the DFD campaign fits with the now extensive body of literature that documents the corporate political strategies of health-harming industries, including transnational alcohol corporations.^4,21,23,26,30-34^ It supports recent studies which focus also on the implementation phase of the policy process as a key aspect of alcohol industry strategy,^34,35^ like that of the tobacco industry before it.^36^ It demonstrates also the potential importance of local-level poltical activity to alcohol- and other health-harming industry actors and the need to inform and protect local government actors, and policy implementation more generally, from industry influence.

 For organisations such as PHE and other government agencies it highlights the potential reputational damage from partnership with bodies linked to the alcohol industry (eg, through Drinkaware’s financial reliance on industry funding) or even those perceived to be. Since this can have a direct impact on the effectiveness of the policy measures they adopt, and the effectiveness with which these can be implemented, perceptions of COI or industry connections may have a tangible impact on the delivery of these organisations’ core activities as well as their reputation for independence.

 In addition, the present study demonstrates the degree to which knowledge and values (ie, on industry involvement in health policy) of front-line, local policy actors tasked with implementing national decisions and campaigns, such as DFD, will influence the uptake of, and the degree of buy-in for, these measures, thereby affecting the degree of support, and likelihood of success, it received. While all LAs face limited resources and industry-funded or associated campaigns may represent additional resources, accepting these may come with a price, and well-informed policy actors are better placed to evaluate these issues and make appropriate decisions. What is evident from the proceeding analysis is that the delivery of the programme, and the degree of promotion it received from LA actors and NGOs, was impacted by the involvement of Drinkaware and the perception among these policy implementers that this was an industry body with a clear COI in its involvement with this programme, and with government agencies such as PHE more generally. From a process perspective policy delivery was at least suboptimal, although where the divide between success and failure lies, and the usefulness of such a binary evaluation, is unclear.

 The limitations of this study included the response rate to the online survey (21%), which was low but comparable to what is often obtained with this type of method.^37-40^ To gain a reliable understanding of policy processes and events,^41^ we triangulated survey findings against the in-depth interview data. There were a relatively high number of respondents responding ‘don’t know’ to certain questions indicating low levels of engagement with the campaign in some quarters. The relatively high proportion of interviewees declining audio recording compared to previous studies undertaken by the authors in the area of alcohol policy reflects the controversial nature of the campaign and the wider policy space. The relatively small number of respondents and lack of sampling procedures mean that the quantitative data presented need to be treated with caution and are designed to be indicative of trends among participants, rather than robust statistical findings which would require a study of larger ‘n’ and the application of different methods and analytical techniques.

## Conclusion

 Given the potential COIs involved in engagement with industry-funded bodies, the above findings provide a cautionary tale for policy-makers and implementers in national and local government faced with dilemmas about whether to partner, or otherwise engage, with industry-associated bodies in the development or delivery of policy measures. Finally, they highlight the need for policy-makers at all levels to develop a more nuanced understanding of industry strategy, the legitimate role for industry-associated bodies in policy-making and delivery and the ways in which influence may be exerted even when such actors present themselves as offering solutions to ongoing policy problems. This will be of relevance to alcohol policy and policy implementation scholars in the United Kingdom and beyond as well as those engaged in policy debates in other areas affected by health-harming industries.

## Acknowledgements

 The authors would like to thank Christopher Turner for his assistance with implementing the online survey questionnaire.

## Ethical issues

 London School of Hygiene and Tropical Medicine (LSHTM) ethics committee.

## Competing interests

 The authors were funded by Public Health England to conduct this study.

## Authors’ contributions

 HW and BH conceived of the study. HW led the writing of the manuscript. All authors were involved in the study analysis, and contributed to critical content and to the drafting the manuscript.

## Funding

 This work was funded by Public Health England.
